# Anatomo-Pathological Study of Ringbone and Spavin1Presented at January meeting of the Keystone Veterinary Medical Association.

**Published:** 1902-03

**Authors:** S. J. J. Harger

**Affiliations:** Philadelphia, Pa.


### THE JOURNAL

OF

### COMPARATIVE MEDICINE AND VETERINARY ARCHIVES.

VOL. XXIII. *MARCH, 1902.* No. 3.

### ANATOMO-PATHOLOGICAL STUDY OF RINGBONE AND SPAVIN AS INDICATED BY EXAMINATION OF PATHOLOGICAL SPECIMENS.[1]

BY S. J. J. HARGER, D.V.M.,
PHILADELPHIA, PA.

THERE is some diversity of opinion as to the manner of production, the seat of the primary lesion, and the order of progression of the successive stages of the alterations of these diseased conditions. I anticipate that some of you may entertain varying views upon these points, and I shall merely interpret the facts as they appear to have presented themselves to me from a series of specimens which I have examined.[2]

*Ringbone.* By this term I shall designate the exostoses having for their base the bones from the middle of the first phalanx to the os pedis (coronary ringbone). It is most frequently the lower extremity of the first and the upper end of the second phalanges—at the tuberosities where the lateral ligaments are attached—that are the primary seat of the exostosis. In rare cases the latter may be confined to the pyramidal eminence of the third phalanx, involving the termination of the anterior extensor tendon of the phalanges and accompanied by a bulging of the superior border of the hoof. The distinction of high and low, true and cartilaginous ringbones of the English and French should, I believe, be discarded.

Ringbones, according to the seat of the lesion, may be articular and periarticular.

In the former the process is as follows: In the compact bone tissue, a short distance under the articular cartilage, a rarefying

[1] Presented at January meeting of the Keystone Veterinary Medical Association.
[2] Specimens in the museum, Veterinary Department, University of Pennsylvania.

osteitis takes place over an area appearing in transverse section as large as a pea or larger. The bone tissue becomes absorbed, leaving a cavity filled up with a more or less soft, embryonic tissue and bloodvessels; in other words, a rarefying osteitis. The process extends gradually toward the articular cartilage, which becomes ulcerated over irregular areas and desquamates, as is frequently seen in cases of osteoporosis, constituting an osteo-arthritis. This inflammatory zone in the bone now becomes the seat of a condensing osteitis (osteosclerosis). The soft, vascular, embryonic material now becomes organized, infiltrated with calcareous matter, and converted into condensed or compact bone. The articular cartilage of the opposing bone at a point opposite to articular ulceration of the bone first affected likewise commences to ulcerate, and leads to an osteo-arthritis at that point. The two articular ulcerations being contiguous will finally form adhesions and become ossified. This centre of ossific union may be localized, or in aggravated cases may involve the entire articular surface and form a complete ankylosis. There is first a rarefying, then a condensing osteitis, and, finally, ossific union of the two bones at those points where the process is completed. The articular ulceration usually commences toward the margin of the articular surface.

The histological alterations are the absorption of the bony tissue and the bone cells, enlargement, and saccular dilatations (lacunæ of Howship) of the Haversian canals, which are filled with embryonic cells and congested bloodvessels—a picture which is repeated in the generalized bony lesions of osteoporosis, and called osteitism or otitic diathesis, which so often in these cases predispose to fracture.

*Peri-articular.* The exostosis develops upon the periphery of the bone. It is an osteoperiostitis. It commences as a rarefying and then passes into a condensing osteitis of the superficial or subperiosteal layers of the compact tissue of the bone, which is communicated to the periosteum and provokes a periostitis. The result is a bone tumor of variable dimensions. The exostoses of the ends of two contiguous bones may become ossified, forming a peripheral or false ankylosis, while the articular surfaces themselves may retain their normal state.

The structure of an exostosis differs slightly from normal bone: It is more porous; the Haversian canals are larger, saccular, and filled with embryonic tissue and bloodvessels; the bone cells are less numerous; its earthy salts are less abundant, and by maceration in an acid solution it becomes decalcified in much less time than normal bone. The ligaments and the synovials also show inflammatory alterations.

*Reciprocal Relation of Articular and Peri-articular Ringbones.* Which is primary and which is secondary, when both exist? Which occurs the more frequently? This is what I more especially sought for. Udriski, of the Bucharest Veterinary School, examined 55 specimens: 20 were exclusively peri-articular, without any articular lesion; 24 showed lesions in both places; 11 were ankylosed. The collection which I examined consisted of 29 specimens: 20, or two-thirds of the entire number, were exclusively peri-articular; 9 showed both external and internal lesions. Among the latter there was ankylosis, partial or complete, and while the exostoses were well marked or large, the articular ossification was progressive—that is, the exostoses were more or less stationary; the only difference in the specimens was the successively increasing degree of the obliteration of the joint, which seems to have been the last thing to have taken place. In one case there was false ankylosis, while the articular surfaces were normal. In no case was there any articular ulceration without periosteal deposit.

The evidence obtained from these specimens seems to point to the general deduction that ringbone begins primarily on the periphery of the bones, and only secondarily extends into the joint. While it cannot be denied that the articular lesions may be primary, these cases are by far in the minority; and in such a case, before the local temperature is altered, a correct diagnosis is difficult, if not impossible.

It would follow from these statements that in prescribing treatment early, and before there is any mechanical interference in the joint movement, the prognosis should, in the majority of cases, be favorable; while, when the articular surfaces are already affected, the prognosis should be reversed. We know that, for old ringbones, firing is not very successful; ankylosis is difficult of production because of the difficulty of destroying so large and deep-seated articular surfaces as those of the coronary joint.

*Spavin.* Opinions are at variance as to whether spavin (arthritis chronica deformans of man) commences peripherally as a periostitis or centrally as an osteo-arthritis of the hock bones. Does the lesion progress centripetally or centrifugally? Most veterinarians are inclined to the former view, and for this purpose I have examined about 40 specimens which have been collected promiscuously at various times. I shall not here refer to the mechanism of the causation of hock diseases.

Upon these specimens the following alterations were observed:

In 7, no exostosis. Occult spavin-ankylosis of the scaphoid and large cuneiform.

Ankylosis scaphoid, large and small cuneiform, in 11.

Ankylosis scaphoid, large and small cuneiform, and metatarsus in 10.

Ankylosis scaphoid, large and small cuneiform, and cuboid in 7.

The remaining were of miscellaneous ossifications, between the astragalus and calcis, astragalus and scaphoid (least frequent), the entire tarsus, etc.

In all cases accompanied by an exostosis the interarticular lesions were well advanced; and in no case excepting one—and this was exceedingly doubtful—was there any peripheral deposit with a normal state of the articular surfaces.

The general order in which, from these observations, the hock bones became affected was as follows: The scaphoid and large cuneiforms, the three internal bones of the lower row, the lower row and the metatarsus, and, finally, the three internal bones of the lower row, with the cuboid of the same row. If I am correct in making these deductions the lesions of spavin should commence as a scaphoid, large, cunean arthritis, and, contrary to the frequent assertion, the small cuneiform was not the seat of the primary lesion. The so-called "occult" spavin may, therefore, be a variety of spavin in its usual sense when the lesions remain in that condition for a longer or shorter time, or merely the beginning of a condition that spreads to other bones and subsequently manifests itself by an external enlargement. The time required for the latter to become visible on the exterior is usually said to be from six weeks to two months. Spavin, therefore, seems to develop eccentrically, beginning within the internal bony structure and the articular surfaces.

We cannot deny the probability of spavin commencing as a periosteitis in cases of traumatisms and hyperextension of the internal ligaments of the hock joint, but this is not the general mode. It also appears to me that these are not the usual causes, but that the latter operate incessantly at every step the animal takes, in the form of constant concussions transmitted through these bones, the perpendicular pressure of one bone upon the other, and the traction of the interosseous ligaments. Relative to inheritance in which there is a defect in the organization and integrity of the intimate structure of bone—an osteitic diathesis—the hock bones are subjected to the injurious effects of these constant causes, and thus become diseased more readily.

Very recent cases of undoubted hock lameness without visible external alterations often respond to the effects of a good blister. Having said that such diseased conditions of the hock are primarily articular, I do not mean to convey the idea that the blister cures by producing ankylosis, such as cauterization does. These cases only present a sort of nerve irritation—the primary symptoms of inflammation without any decided structural changes—and the treatment is efficacious through its revulsive action, a possible reflex action through the nerve trunks in correcting the pain and the circulation, and the immobilization of the parts.

From a comparison of the external and internal lesions of ringbone and spavin we find the conditions in the two reversed, the former developing from without to within, and the latter from within to without, the enlargement of the hock being secondary. While some of the distinctions which I have here made may not be mathematically as accurate as you may be led to believe, they appear sufficiently conclusive to warrant the general deductions and to form a conception of the pathology.

Chronic arthritis of the tarsus seems to be more amenable to treatment—ankylosis—with the actual cautery than the same lesion on the phalanges. The cautery can be brought in more direct contact with the seat of the disease; the bones of the hock are porous and vascular; the articular surfaces are covered by a thin layer of articular cartilage and probably more readily exfoliated; the bones are almost immovable and in very close apposition, and have a paleontological tendency to diminution from ossification and a disappearace of the individual bones of the anatomical foot. The last fact may be pertinent from a medico-legal point of view. I read an account of this kind in the *Revue Veterinaire*. The subject was a young mare. When the bones were cleaned several of the hock bones were neatly ossified, although at no time during life, so far as could be learned, was there any knowledge of lameness. I have seen such an ossification of the astragalus and the os calcis in the hock of a horse in spite of the gliding movement that exists between these two bones, and no deformity could be seen on the surface. I know nothing about the animal from which it was obtained. Under any circumstances, a lameness, even though temporary, may accompany such a condition, and we are at sea when we endeavor to locate it.

